# Multiple monoaminergic modulation of posturo-locomotor network activity in the newborn rat spinal cord

**DOI:** 10.3389/fncir.2014.00099

**Published:** 2014-08-15

**Authors:** Lauriane Beliez, Gregory Barrière, Sandrine S. Bertrand, Jean-René Cazalets

**Affiliations:** CNRS UMR 5287, Institut de Neurosciences Cognitives et Intégratives d’Aquitaine, Université de BordeauxBordeaux, France

**Keywords:** dopamine, serotonin, noradrenaline, locomotion, posture, spinal cord, neuromodulation

## Abstract

Studies devoted to understanding locomotor control have mainly addressed the functioning of the neural circuits controlling leg movements and relatively little is known of the operation of networks that activate trunk muscles in coordination with limb movements. The aim of the present work was (1) to identify the exogenous neurotransmitter cocktail that most strongly activates postural thoracic circuitry; (2) to investigate how the biogenic amines serotonin (5-HT), dopamine (DA), and noradrenaline (NA) modulate the coordination between limb and axial motor networks. Experiments were carried out on *in vitro* isolated spinal cord preparations from newborn rats. We recorded from ventral roots to monitor hindlimb locomotor and axial postural network activity. Each combination of the three amines with excitatory amino acids (EAAs) elicited coordinated rhythmic motor activity at all segmental levels with specific characteristics. The variability in cycle period was similar with 5-HT and DA while it was significantly higher with NA. DA elicited motor bursts of smaller amplitude in thoracic segments compared to 5-HT and NA, while both DA and NA elicited motor bursts of higher amplitude than 5-HT in the lumbar and sacral segments. The amines modulated the phase relationships of bursts in various segments with respect to the reference lumbar segment. At the thoracic level there was a phase lag between all recorded segments in the presence of 5-HT, while DA and NA elicited synchronous bursting. At the sacral level, 5-HT and DA induced an intersegmental phase shift while relationships became phase-locked with NA. Various combinations of EAAs with two or even all three amines elicited rhythmic motor output that was more variable than with one amine alone. Our results provide new data on the coordinating processes between spinal cord networks, demonstrating that each amine has a characteristic “signature” regarding its specific effect on intersegmental phase relationships.

## INTRODUCTION

The achievement of efficient locomotory movements necessary for survival in a demanding external environment requires the integrated functioning and synergistic action of many muscle groups in order to ensure the appropriate positioning of all body regions during self-motion (Cazalets, 2000; Falgairolle et al., 2006). However, most studies devoted to understanding locomotor control in humans and quadrupeds (Rossignol, 1996; Duysens and Van de Crommert, 1998) have only addressed the functioning of the neural circuits controlling leg movements, and relatively little is known about the functioning of neuronal networks that activate trunk muscles in coordination with limb movements (Thorstensson et al., 1982; Koehler et al., 1984; Zomlefer et al., 1984; Gramsbergen et al., 1999; De Sèze et al., 2008; Rauscent et al., 2009; Combes et al., 2012). Similarly, the role of the forelimbs during locomotion remains poorly understood (Akazawa et al., 1982; Duenas et al., 1990; Ballion et al., 2001; Zehr and Duysens, 2004). Indeed all these motor networks do not operate in isolation but interact with each other, and it is only recently that increasing attention has been paid to the interactive functioning of various spinal segments in quadrupedal mammals and humans during locomotion (Cazalets, 2000; Wannier et al., 2001; Dietz, 2002; Falgairolle et al., 2006, 2013; Maclellan et al., 2011; Nakajima et al., 2013).

Several seminal reviews (Harris-Warrick, 2011; Miles and Sillar, 2011) have recently highlighted the role of neuromodulators in governing the functional flexibility of spinal circuitry. The motor networks responsible for locomotor activity and which are the potential targets of neuromodulators are intrinsic to the spinal cord and can generate rhythmic motor activity in the absence of sensory feedback. The cervical and lumbar enlargements contain the neural machinery necessary to operate the fore- and hind-limbs, respectively, while thoracic and sacral segments control the axial systems. As underlined by Harris-Warrick (2011), neuromodulatory actions are required for the normal function of these networks. In using the *in vitro* isolated spinal cord from new born rat, the neuromodulatory impact of the three biogenic amines serotonin, dopamine (DA), and noradrenaline (NA) on locomotion has been extensively studied. Their effect had been previously established for the motor activity generated by the lumbar segments and subsequent studies further investigated aspects of their pharmacological features (Cazalets et al., 1992; Kiehn and Kjaerulff, 1996; Kiehn et al., 1999; Sqalli-Houssaini and Cazalets, 2000; Barriere et al., 2004; Madriaga et al., 2004; Pearlstein et al., 2005; Gordon and Whelan, 2006; Liu et al., 2009). It has been shown that they may differentially modulate a motor pattern according to the specific receptor subtypes they activate (for review, see Miles and Sillar, 2011). 5-HT exerts an excitatory (permissive) effect through an activation of 5-HT2 and 5-HT7 receptors (Madriaga et al., 2004; Pearlstein et al., 2005; Liu et al., 2009) or an inhibitory (suppressive) action through 5-HT1 receptor activation (Beato and Nistri, 1998) on motoneurons or interneurons (Zhong et al., 2006a). Similarly, NA (Kiehn et al., 1999; Sqalli-Houssaini and Cazalets, 2000), exerts a multi-modal control of lumbar locomotor networks through an α1 receptor mediated activation and an α2 receptors mediated inhibition (Sqalli-Houssaini and Cazalets, 2000; Gabbay et al., 2002; Gordon and Whelan, 2006). The dopaminergic modulation of lumbar locomotor patterns (Kiehn and Kjaerulff, 1996; Whelan et al., 2000; Madriaga et al., 2004) is mediated via D1 and D2 receptor activation leading to an increase in motoneuron and Hb-9 interneuron excitability (Han et al., 2007). Most of the effects reported for these drugs were also found to occur in spinally transected adult rodents (Landry et al., 2006; Lapointe and Guertin, 2008; Lapointe et al., 2008, 2009). Surprisingly, however, despite the numerous studies that have investigated the individual action of each amine alone or in combination with excitatory amino acids (EAAs), there has not been a study that has systematically compared the action of the three amines, alone or in various combinations, on locomotor pattern genesis.

The aim of the present study therefore was (1) to identify the neuromodulator mixture that most strongly activates spinal motor networks involved in hindlimb and axial muscle control; (2) to investigate how the biogenic amines serotonin (5-HT), DA, and NA, shape the operation of and the interactions between the limb and axial motor networks during ongoing locomotor-like rhythmogenesis initiated by excitatory amino-acid receptor activation.

## MATERIALS AND METHODS

Experiments were performed on the *in vitro* isolated spinal cord of newborn Sprague–Dawley rats aged between 1 and 5 post-natal days (P1–P5,* n* = 25 preparations). All procedures were conducted in accordance with the local ethics committee of the University of Bordeaux and the European Committee Council Directive. All efforts were made to minimize animal suffering and to reduce the number of animals.

### *IN VITRO* ISOLATED SPINAL CORD

Rat pups were anesthetized using isofluorane until no reflex could be elicited in response to tail or toe pinching. Animals were decapitated, the skin of the back removed, and the preparations were placed ventral side up in a dissecting chamber. A laminectomy was performed to expose the spinal cord that was then dissected out using fine forceps and microscissors under binocular microscope control. Dissections and recording procedures were performed under the continuous perfusion of an artificial cerebrospinal fluid (aCSF) equilibrated with 95% O_2_–5% CO_2_, pH7.4 at room temperature (24–26^∘^C) and containing (mM): NaCl 130, KCl 3, CaCl_2_ 2.5, MgSo_4_ 1.3, NaH_2_PO_4_ 0.58, NaHCO_3_ 25 and glucose 10. Spinal cords were sectioned at the T2 level at the beginning of the experiment.

### ELECTROPHYSIOLOGICAL RECORDINGS AND ANALYSIS

Extracellular motor activities were recorded from various spinal cord ventral roots using Vaseline-insulated, stainless steel pin electrodes at the lumbar level and glass suction electrodes for the shorter thoracic ventral roots. Recorded activities were amplified using custom-made amplifiers then digitized (Digidata 1322A, Molecular Device, CA, USA) using an interface driven by Axograph software (Axograph, AU). The raw signals were high-passed (50 Hz), rectified and integrated before analysis. Burst amplitudes (trough to peak) and locomotor pattern cycle periods were calculated using a program developed in Matlab (Mathworks) based on burst onset and offset detection. Since it is not possible to compare absolute amplitude values of extracellular data between experiments, we calculated only the relative change in amplitude from a control condition. Such amplitude changes could be either due to changes in firing frequency of already active motor units or to a recruitment of previously silent units. Due to the difficulty in establishing stable and persistent rhythmic locomotor-like activity with bath-application of EAAs alone, the combination of EAAs and 5-HT that is the most commonly used cocktail to induce locomotor-related activity *in vitro* (Sqalli-Houssaini et al., 1993) was taken as the reference condition to compare the actions of the amines.

We used recordings at L2 as the reference trace because it invariably exhibited the best signal-to-noise ratio. Pair-wise phase relationships between bursting activities recorded simultaneously from different ventral roots were calculated using wavelet transform analysis (for an extensive description of the method see Grinsted et al., 2004; Mor and Lev-Tov, 2007; Torrence and Compo, 2010). For this purpose, we used custom-made software based on the MatLab wavelet coherence package provided by Aslak Grinsted (http://noc.ac.uk/using-science/crosswavelet-wavelet-coherence). For each pair-wise recording we performed both a cross wavelet transform and a wavelet coherence determination. The results obtained with both algorithms were combined into a single time-frequency map so as to extract phase relationships from delineated coherent common high power frequency regions. Phase values were plotted as a circular representation (0–1 rad), with the mean phase being indicated by the direction of the vector, and its length (range from 0 to 1) indicating the strength of the mean.

### PHARMACOLOGY

Episodes of locomotor-like activity were elicited by the exogenous application of a mixture of 15 μM N-methyl-D,L-aspartate (NMA, Sigma) and serotonin (5-HT, Sigma) and/or DA (Sigma), and/or NA (Sigma). DA and NA were freshly prepared and protected from light exposure during bath superfusion in order to prevent photo-degradation. Sodium metabisulfite (0.1%; Sigma) was also added to the DA supply in order to prevent oxidation (Barriere et al., 2004). Drugs were bath-applied using a peristaltic pump (flow rate 4 ml/min; recording chamber volume 4 ml). The effects of the drugs were monitored from 5 to 10 min after reaching the Petri dish (the time required for total replacement of the normal saline and diffusion into the tissue).

Different drug combinations were tested on the same preparation in a random order except for DA. Since we previously demonstrated that high DA concentrations (500 μM) may exert long-lasting effects on the *in vitro* newborn rat spinal cord (Barriere et al., 2004), this amine was always tested after the other amines in a given experiment and at a 10 times lower concentration than that found to exert prolonged actions (i.e., 50 μM instead of 500 μM). When different drug concentrations were tested in the same preparation, we always began with the lowest concentration. Total drug application lasted 20 min. During an episode of locomotor-like activity, cycle periods were initially longer and then shortened progressively until stable rhythmicity was reached within 5 min (Sqalli-Houssaini et al., 1993; Cazalets et al., 1999). All measurements were performed on 30 consecutive cycles during this steady-state condition. Each drug bath-application was followed by a prolonged wash out with normal saline for at least 30 min.

### STATISTICAL ANALYSIS

Data were tested for normality, and in its absence, non-parametric analysis was applied. Statistical analyses were performed using Prism software (Graphpad software, CA, USA). The coefficient of variation (COV), known as “relative variability,” was determined by the SD divided by the mean and expressed as a percent. In each experiment it was calculated from the same recording sequence as the motor period and amplitude. The degree of variability (or stability) of the motor pattern cycle period was assessed by computing the COV. Comparisons between conditions were performed using Student’s *t*-test, one-way or two-way ANOVA with Tukey’s test *post hoc* analysis. The equality of group variance was tested using the Bartlett’s test or the *F* test.

Statistical analyses of circular data were performed on raw data by descriptive statistics of circular distribution using IgoPro software (Wavemetrics, OR, USA) and Oriana software (KCS, UK). The Rayleigh test was used to determine the coupling strength. The Watson–Williams test (a circular analog of the one-factor ANOVA, Berens, 2009) and a non-parametric second-order two-sample test (which is less sensitive to departure from normality) consisting of pre-processing where the grand mean is subtracted from the two inputs followed by application of Watson’s *U*2-test (IgoPro software, Wavemetrics, OR, USA) were used.

The significance threshold was taken to be *p* < 0.05 unless otherwise specified. All data values in the text are means ± SEM.

## RESULTS

**FIGURE 1:**
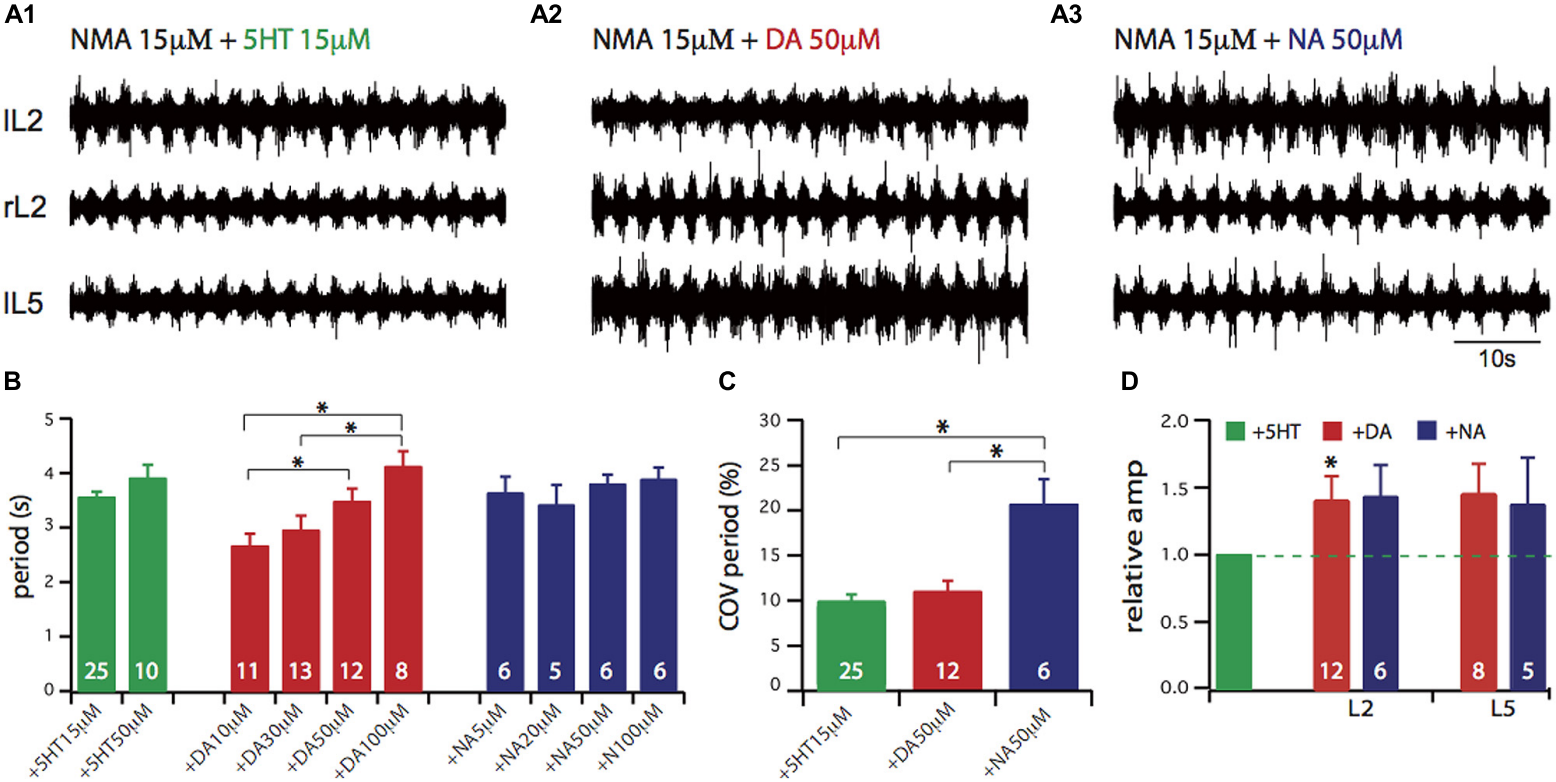
**Comparison of lumbar locomotor network activation *in vitro* by 5-HT, or DA or NA. (A)** Each amine was added to the bathing saline containing the NMDA excitatory amino acid receptor agonist NMA (15 μM), while extracellular recordings were made from the second left and right lumbar ventral root (lL2, rL2) and the fifth left lumbar ventral root (lL5) of the isolated spinal cord. **(B)** Plots of mean cycle period value obtained for different amine concentrations. A dose-dependent effect is only observable for DA. **(C)** Plots of the coefficient of variation (COV) of cycle period for each amine (NMA 15 μM, 5-HT 15 μM, DA 50 μM, NA 50 μM), indicating that NA leads to a more unstable motor activity. **(D)** Plots of mean relative amplitude changes normalized to the amplitude observed in the NMA/5-HT condition (green bar). DA and NA elicit larger burst amplitude in the lumbar ventral roots. In this and in subsequent figures, the number on each bar histogram indicates the number of experiments. ^∗^*p* < 0.05.

Since no direct comparison of the neuromodulatory action of the three monoamines 5-HT, NA and DA has been so far reported, we first established how they each modulate the basic rhythmic motor activity elicited by EAAs. For this purpose we recorded ipsi- and contra-lateral L2 and L5 lumbar ventral roots to respectively monitor the flexor and extensor phases of the motor pattern cycle (**Figures 1A1–A3**). From a given experiment we selected representative episodes of locomotor-like activity with comparable cycle periods elicited by the conjoint bath-application of the EAA agonist NMA and one amine in order to highlight possible differences between other parameters (such as COV, phase relationship, and motor burst amplitude). Several parameters that characterize the ongoing locomotor patterned activity were then calculated, namely the cycle period (the time that separates the onsets of two successive bursts of activity, **Figure 1B**), the COV of cycle period which is an index of the temporal regularity of the motor pattern (**Figure 1C**) and the burst amplitude (**Figure 1D**). As previously found (Sqalli-Houssaini and Cazalets, 2000; Barriere et al., 2004), there was a significant dose-dependent action for DA on cycle period (one-way ANOVA, *F* = 6.06, *p* = 0.0016) but not for NA (one-way ANOVA, *F* = 0.8, *p* = 0.5) and 5-HT (Student’s *t*-test, *t* = 1.53, *p* = 0.14), over the range of concentrations tested. In the present study, subsequent experiments were performed using DA at 50 μM, an intermediate concentration in the dose-dependent range. When comparing the variance of cycle period in the presence of the three amines at the concentrations used in **Figure 1A** (and throughout the study), we found that values were significantly different (Bartlett’s test for equal variance, Bartlett’s statistics = 10.5, *p* = 0.005). This variability in cycle period revealed by the COV calculation (**Figure 1C**), was comparable for NMA/5-HT and NMA/DA (9.3 ± 1.3% and 11 ± 2%, respectively) while it was significantly higher for NMA/NA (20.7 ± 2.8%, one-way ANOVA, *F* = 10.5, *p* = 0.0002). For each amine, there was no evident dose-dependency for period variability. **Figure 1D** shows the relative changes in motor burst amplitude. For each trial, the mean burst amplitude of activity in an individual ventral root under NMA/5-HT was considered as the control condition and measurements under other pharmacological conditions were normalized to this value. Both DA and NA significantly increased the amplitude of flexor (L2) and extensor (L5) motor bursts. Altogether, these results confirm the previously reported individual effects of the amines on EAA-induced locomotor-related rhythmicity, although our comparative assessment indicates that each amine exerts a specific action on hindlimb motor network activity.

**FIGURE 2:**
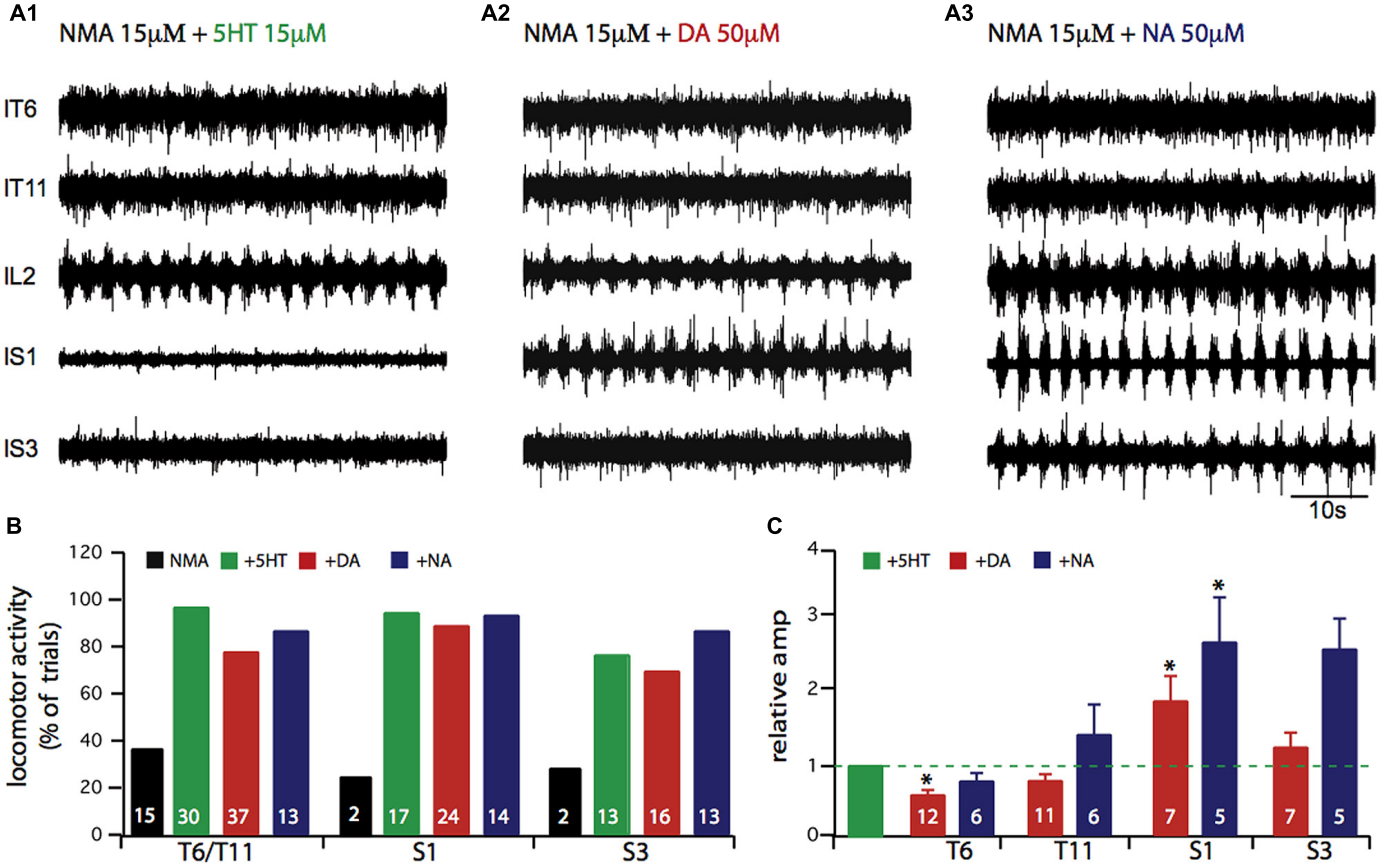
**Comparison of axial locomotor network activation *in vitro* by 5-HT, or DA or NA. (A)** Each amine was added to 15 μM NMA-containing saline during recordings from the sixth and eleventh left thoracic ventral roots (lT6, lT11), the second left lumbar ventral root (lL2), and the first and third left sacral ventral roots (lS1, lS3). **(B)** Plots indicating the percentage of experiments in which rhythmic motor activity was observed at a given segmental level. In these experiments, lumbar locomotor activity was always expressed. **(C)** Plots of mean relative amplitude changes normalized to the amplitude observed in the NMA/5-HT condition (green bar). Note the higher noradrenergic sensitivity of the more distal segments. ^∗^*p* < 0.05.

In a next step, we tested which amine most strongly activates the axial and hindlimb motor networks. **Figure 2A** presents typical simultaneous recordings from thoracic (T6 and T11), lumbar (L2) and sacral (S1, S3) ipsilateral ventral roots obtained from the same experiment in the presence of NMA and successively 5-HT (A1), DA (A2), and NA (A3) at amine concentrations that elicited motor rhythms with similar cycle periods. Each amine elicited coordinated motor burst at all segmental levels, although with qualitative differences. Visual trace inspection suggests that the most striking changes took place in the sacral burst amplitude in the presence of DA and especially NA. In the thoracic region, motor bursts were more distinguishable in the presence of 5-HT than either DA or NA. **Figure 2B** indicates the proportion of experiments in which motor bursts were recorded from thoracic and sacral segments when sustained lumbar locomotor-like activity was elicited as defined in **Figure 1**. In this respect, 5-HT and NA were noticeably more effective than DA. Moreover, relative amplitude calculations indicate that burst amplitudes in thoracic segments were significantly decreased by DA (**Figure 2C**; paired Student’s *t*-test, *t* = 5.4, *p* = 0.0002), while burst amplitude in the sacral segments was significantly increased by DA (**Figure 2C**; paired Student’s *t*-test, *t* = 2.8, *p* = 0.03) and NA (**Figure 2C**; Student’s *t*-test, *t* = 4.5, *p* = 0.006).

**FIGURE 3:**
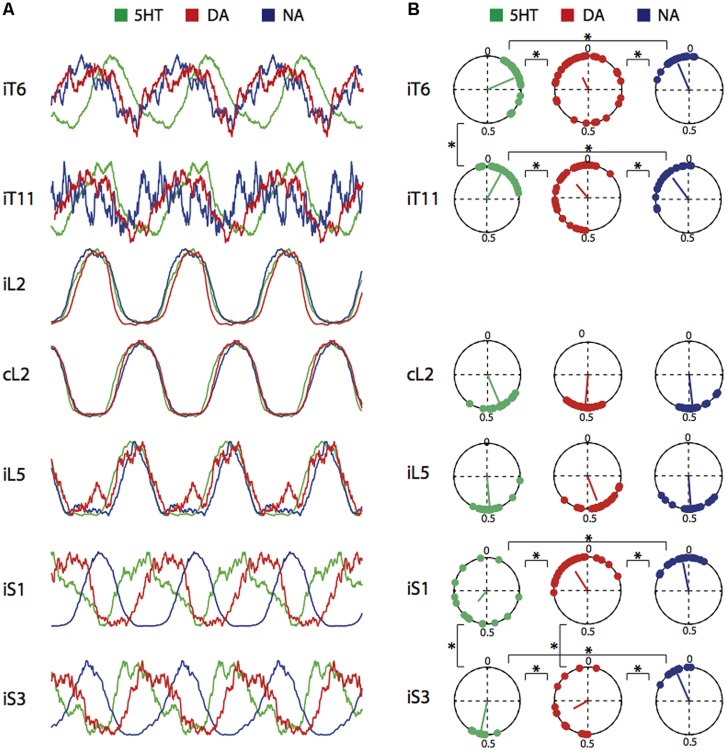
**Control of phase relationships by 5-HT, DA, or NA. (A)** Averaged and replicated cycle sequences during locomotor-like activity induced by a combination of NMA (15 μM) and one of the three amines (5-HT, 15 μM; DA, 50 μM; NA, 50 μM). Bursts were normalized for the amplitude. Note phase differences relative to reference L2 bursts shift occurring in the thoracic and sacral segments. **(B)** Circular plots showing the mean phase values for burst activity in the six ventral roots recorded in A relative to the L2 ventral root in the presence of each three amines. Each point is the phase value measured from one experiment. The vector direction represents the grand mean phase value and its length is a function of the coupling strength. Data were pooled from all concentrations tested. i; ipsilateral; c, contralateral. ^∗^*p* < 0.05.

**FIGURE 4:**
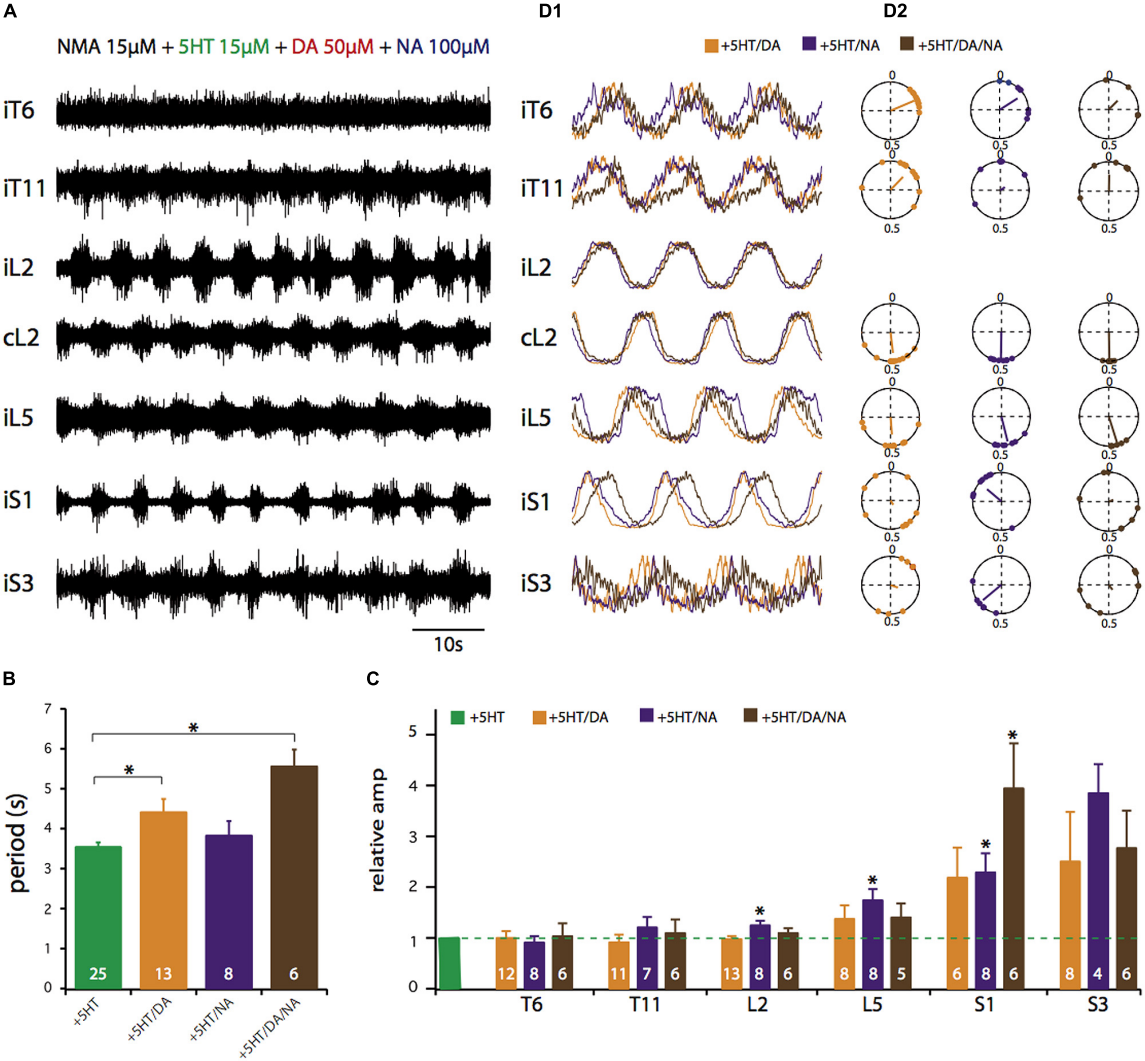
**Control of activity phase relationships by various combinations of NMA and 5-HT/DA/NA. (A)** Simultaneous recordings of seven ventral roots in the presence of all four drugs. **(B)** Plots of mean cycle periods under various drug combinations. **(C)** Plots of mean relative amplitude changes normalized to the amplitude observed in the NMA/5-HT condition (green bar). **(D1)** Averaged and replicated cycles during locomotor-like activity induced by a combination of NMA (15 μM) and the three amines in various combinations. **(D2)** Circular plots calculated from the recordings in **D1** showing the mean phase values for the six ventral roots relative to L2 in the presence of NMA and the different amine cocktails. All data presented were obtained at the following drug concentrations: NMA, 15 μM; 5-HT, 15 μM; DA, 50 μM; NA, 50 μM. ^∗^*p* < 0.05.

Since the combination of NMA and the amines elicited coordinated rhythmic motor activity in the various spinal cord areas, we examined whether, in addition to their effects on cycle period and burst amplitude, they were able to shape locomotor-related activity by modulating the phase relationships between the various segmental levels. Phase relationships were calculated by taking the ipsilateral L2 ventral root recording as the reference trace (see Materials and Methods). **Figure 3A** presents averaged individual cycles replicated in a continuous sequence to reveal the rhythmic nature of the activity (see Figure 6A in Falgairolle and Cazalets, 2007), in the presence of each of the three amines. Each time a burst was detected in the reference L2 trace, it was sampled with the other associated recorded traces. Individual cycles were superimposed and then averaged. This process allowed switching from a continuous mode of recording to an episodic one (**Figures 3A** and **4B**) so as to increase the signal-to-noise ratio in order to obtain a more accurate detection of burst peaks (Falgairolle and Cazalets, 2007). None of the amines elicited significantly different phase relationships in the lumbar left/right alternating pattern, the flexor and extensor coordination nor burst durations, as indicated by the strict superimposition of L2 and L5 traces under all three amine conditions. In contrast, each amine established a distinct phase relationship between the thoracic, sacral and lumbar segments. **Figure 3B** illustrates the phase relationships between each recording location and reference L2. Data were pooled for all concentrations tested as there was no dose-dependent effect of concentration for any amine and any ventral root (multisample Watson–Williams test, *p* > 0.1). Each point in the polar plots represents the mean phase value obtained in a single experiment, with the vector direction representing the mean phase value and its length the coupling strength. Inspection of the polar plots reveals that with 5-HT and DA, the phase values were more variable between experiments in the sacral region than in the lumbar or thoracic segments. In the presence of 5-HT there was a phase lag between lumbar and thoracic segments that was previously shown to increase significantly with the intervening distance (Falgairolle and Cazalets, 2007), while almost synchronous bursting occurred in the recorded thoracic segments with no phase lag increase with segmental distance in the presence of DA (mean vector value 0.93 in T6 and 0.88 in T11; Mardia’s two sample *U* 0.08, *p* > 0.2, **Figure 3**) and NA (mean vector value 0.94 in T6 and 0.89 in T11; Mardia’s two sample *U* 0.07, *p* > 0.2, **Figure 3**). At the sacral level, a significant progressive phase shift relative to L2 occurred both in the presence of 5-HT (mean vector value 0.61 in S1 and 0.52 in S3; Mardia’s two sample *U* 0.24, *p* < 0.02, **Figure 3**), and DA (mean vector value 0.91 in S1, 0.61 in S3; Mardia’s two sample *U* 0.3, *p* < 0.005, **Figure 3**). In contrast, NA elicited a phase-locked motor pattern with a similar phase relationship at all sacral levels (mean vector value 0.97 in S1 and 0.93 in S3; Mardia’s two sample *U* 0.18, *p* > 0.05, **Figure 3**). These results therefore demonstrate that each amine possesses a specific instructive “signature” regarding its modulatory affect on the distributed temporal aspects of the motor pattern.

Finally, we investigated different neuromodulatory combinations by mixing EAA receptor agonists with two or even the three amines (**Figure 4**). A typical experiment in which the four drugs were added to the bath saline is shown in **Figure 4A**. This neuromodulatory cocktail still elicited rhythmic motor output that, at a first glance, did not differ markedly from the condition with one amine alone. However, rhythmic bursting was less frequently observed (67% of experiments, **Table 1**), in the thoracic compartment under the multiple amine condition. Moreover, the amines in combination elicited motor rhythms with significantly longer cycle periods (**Figure 4B**; unpaired Student’s *t*-test; 5-HT/DA, *t* = 2.96, *p* = 0.0057; 5-HT/DA/NA, *t* = 6.82, *p* = 0.0001) and increased burst amplitude in the most caudal (L5 and sacral) segments (**Figure 4C**; paired Student’s *t*-test; 5-HT/NA in L2, *t* = 2.5, *p* = 0.04; 5-HT/NA in L5, *t* = 2.5, *p* = 0.04; 5-HT/NA in S1, *t* = 4.8, *p* = 0.002; 5-HT/NA/DA in S1, *t* = 3.5, *p* = 0.017). The rhythm stability, as indicated by the COV was within the same range as values with the mixture of NMA/5-HT or NMA/DA (see **Figure 1C**), i.e. 10.4 ± 1.2% for NMA/5-HT/DA, 13 ± 2% for NMA/5-HT/NA and 12.6 ± 3.4% for NMA/5-HT/DA/NA. Moreover, phase analysis (**Figure 4D**), revealed that the flexor/extensor relationship remained extremely stable under all conditions (see also **Figure 3B**). In contrast, and especially for the sacral segments, the phase relationships generally became more variable with a very small vector length. Interestingly, however, in the mid-thoracic segment (T6), the phase relationships observed in the presence of all three amines or 5-HT/DA or 5-HT/NA was close to that observed in the presence of 5-HT alone (i.e., at about 0.19; see **Table 2**).

**Table 1:** Summary of the effects of 5-HT, DA and NA.

	Episode duration	Period (s)	Period variability (%)	Relative Amplitude			Presence of motor bursts (%)		
				Thoracic	Lumbar	Sacral	Thoracic	Lumbar	Sacral
NMA		3.04 ± 0.5							
NMA/5-HT	All bath-application	3.55 ± 0.10	9.30 ± 1.34	1	1	1	98	100	76
NMA/DA 50μM	All bath-application	3.47 ± 0.24	11.04 ± 1.17	0.66 ± 0.06	1.40 ± 0.18	1.54 ± 0.21	89	100	79
NMA/NA 50μM	All bath-application	3.8 ± 0.16	20.68 ± 2.76	1.18 ± 0.29	1.22 ± 0.17	2.68 ± 0.45	90	100	100
NMA/5-HT/DA	All bath-application	4.41 ± 0.32	10.38 ± 1.79	0.96 ± 0.10	0.97 ± 0.06	2.35 ± 0.57	85	100	78
NMA/5-HT/NA	All bath-application	3.82 ± 0.36	13.03 ± 1.00	1.04 ± 0.11	1.24 ± 0.09	2.81 ± 0.37	81	100	83
NMA/5-HT/DA/NA	All bath-application	5.56 ± 0.42	12.65 ± 3.40	1.06 ± 0.17	1.09 ± 0.09	3.35 ± 0.57	67	100	92

*Note that the category “episode duration” indicates that a given neuromodulator cocktail induced rhythmic motor activity throughout bath-application.*

**Table 2:** Phase and mean vector length (*r*) value for burst activity in the six ventral roots (**Figure 4D2**) relative to the L2 ventral root in the presence of various combinations of NMA and 5-HT/DA/NA.

	iT6	iT11	cL2	iL5	iS1	iS3
	Phase (*n*) R	Phase R	Phase R	Phase R	Phase R	Phase R
NMA/5-HT	0.19 (31) 0.86	0.08 (31) 0.88	0.43 (28) 0.93	0.49 (18) 0.91	0.61 (15) 0.41	0.53 (9) 0.92
NMA/DA	0.93 (39) 0.40	0.89 (45) 0.54	0.51 (47) 0.94	0.44 (38) 0.80	0.91 (25) 0.67	0.67 (13) 0.49
NMA/NA	0.94 (21) 0.85	0.90 (23) 0.794	0.48 (26) 0.88	0.49 (23) 0.87	0.97 (21) 0.86	0.93 (13) 0.89
NMA/5-HT/DA	0.19 (11) 0.90	0.12 (12) 0.63	0.48 (12) 0.71	0.49 (10) 0.61	0.41 (8) 0.18	0.30 (6) 0.27
NMA/5-HT/NA	0.16 (5) 0.74	0.16 (8) 0.19	0.50 (8) 0.92	0.46 (8) 0.89	0.86 (8) 0.66	0.64 (5) 0.84
NMA/5-HT/DA/NA	0.12 (3) 0.42	0.00 (5) 0.56	0.50 (6) 0.97	0.45 (5) 0.95	0.19 (6) 0.16	0.40 (6) 0.21

## DISCUSSION

### COMBINATORY ACTION OF DRUGS ON MOTOR RHYTHM GENERATION AND STABILITY

As pointed out by Guertin (2009) there is a need in the field of spinal cord injury and recovery for elaborating therapeutic strategies based on drug-induced CPG activation. Hence the identification of candidate molecules that could become first-in-class treatments for spinal cord injured patients in animal models becomes a prerequisite. The first objective of the present work was therefore to determine which neuromodulator cocktail most effectively activates spinal motor networks involved in locomotion. In pioneering studies (Kudo and Yamada, 1987; Smith and Feldman, 1987), the EAA receptor agonist NMDA was reported as the first molecule to evoke locomotor-like episodes in the isolated neonatal rat spinal cord. Later on, we established 5-HT as one of the main actors in locomotor network activation in rodents (Cazalets et al., 1990, 1992), and discovered that the combined bath-application of 5-HT and EAA agonists was a very effective way to reliably elicit sustained episodes of locomotor-like activity (Sqalli-Houssaini et al., 1991, 1993). Since then, it has become the most widely used method to eliciting locomotor-like activity in this preparation, and hundreds of reports have been released based on this seminal observation. In contrast, only few studies have documented the combined activating action of EAA/DA or EAA/DA/5-HT (Kiehn and Kjaerulff, 1996; Jiang et al., 1999; Whelan et al., 2000; Juvin et al., 2005; Zhong et al., 2007; Talpalar and Kiehn, 2010), or EAA/NA (Kiehn et al., 1999; Sqalli-Houssaini and Cazalets, 2000), and in each case there was no clear experimental rational for employing this combined EAA/amine approach. Surprisingly, despite the outstanding opportunity provided by the use of an *in vitro* preparation, no study has systematically addressed the issue of combined drug actions and no comparative study of permissive neuromodulator interactions is available. Although some idea of the influences of these amines together can be gleaned indirectly from the literature, the disparity in protocols used (saline composition, temperature, rat strain,…) has prevented a direct comparison to be made. Furthermore in most studies, motor output from only the lumbar cord region was investigated.

An important prerequisite is to clearly define the limits and the terms of what can be considered as the “optimal” form of locomotor output *in vitro*. There is a general consensus in the field that spinally generated rhythmic motor activity is considered as “locomotor-like” when alternating flexor and extensor bursts are recorded extracellularly from the L2 and L5 ventral roots (Cazalets et al., 1992; Kiehn and Kjaerulff, 1996) and when the pattern cycle period falls within a range similar to that observed in the intact animal at the same age (1s; Cazalets et al., 1990; Jamon and Clarac, 1998). However, caveats in the respect include the effect of temperature on the *in vitro* rhythm frequency, since from 25^∘^C (the typical mean room temperature used in most *in vitro* studies) to 35^∘^C (the usual pup body temperature and the temperature at which *in vivo* experiments are performed (Cazalets et al., 1990), the cycle period decreases by more than 50% (Sqalli-Houssaini et al., 1991). A similar observation has been reported for saline K^+^ concentration that is frequently twice the normal concentration (3 mM; Somjen, 1979) measured in the intact animal. As such, the “best” *in vitro* motor output pattern would have a cycle period of ∼2–3 s (when taking into account the slowing down of the motor period resulting from the isolated CNS conditions), with fictive locomotor episodes lasting for the duration of drug bath-application (i.e., without receptor desensitization or any other processes that would prematurely curtail rhythmogenesis) and ventral root bursts that, in a given drug condition, occur as regularly as possible and with little variation both in their amplitude and inter-root phase relationships. Based on these premises, **Table 1** summarizes the data collected in the present and previous studies. It includes data of thoracic, lumbar and sacral motor output and under exposure to various drug combinations. A comparison of the compiled values indicates that there is no optimal way to elicit locomotor-like activity *in vitro* since several neurotransmitter combinations are able to elicit stable rhythmicity with *in vitro* locomotor compatible periods and phase-relationships. For example, motor burst amplitude is evidently more sensitive to NA, whereas rhythm stability is provided by DA or 5-HT. Presumably such differences indicate that the various parameters of the spinal motor system can be separately adjusted according to changing behavioral requirements. Nevertheless, it remains apparent that the combination of EAA/5-HT constitutes a good compromise in terms of rhythm stability, burst amplitude, and the recruitment of the different spinal compartments. However, specifically for sacral activity, it would be perhaps more interesting to use NA or DA which elicit higher burst amplitudes. To date it is not possible to determine the location at which the neuromodulatory processes described here may occur. With increasing age, there is a decrease in the motor period variability in the intact animal (Cazalets et al., 1990). Both the bath-application technique used here and the developmental changes undergone by neuromodulatory pathways themselves do not allow us to draw any conclusion about the precise functional role played by each amine in the intact adult animal.

Rhythmic motor activity elicited by the conjoint bath-application of EAA agonists and the three amines separately or in combination always led to locomotor pattern genesis (**Figures 1** and **4**). Our previous studies have already described the pharmacological action of the three amines and how they modulate NMA-induced locomotor-like activity (Sqalli-Houssaini et al., 1993; Sqalli-Houssaini and Cazalets, 2000; Barriere et al., 2004). In the present study, the same NMA concentration was the common parameter for all conditions, thus allowing direct comparison to be made between the different amine actions. Of the three amines, however, only 5-HT triggered activity that corresponded most closely to actual locomotion in terms of cycle period and inter-segmental phase relationships. In contrast, neither NA nor DA alone evokes locomotor-compatible rhythms, the latter in terms of cycle periods (Kiehn and Kjaerulff, 1996; Barriere et al., 2004) and the former in terms of cycle periods and flexor/extensor phase relationships (Kiehn et al., 1999; Sqalli-Houssaini and Cazalets, 2000; Gabbay and Lev-Tov, 2004). On this basis, therefore, it could be that the locomotor signature of spinal output rhythmicity is provided mainly by EAAs since the activity they elicit possess the closest parameters to the cycle periods and flexor and extensor alternation observed *in vivo* (Kudo and Yamada, 1987; Cazalets et al., 1992). In such a framework, EAAs could establish basal temporal features of the motor rhythm with a cycle period that varies according to the amount of endogenous EAA released (Kudo and Yamada, 1987; Cazalets et al., 1992; Beato et al., 1997; Talpalar and Kiehn, 2010). During real CPG operation, the main task of the amines would be to modulate this “primary” EAA-induced pattern via their combinatorial release.

To date, the cellular mechanisms by which the amines exert their effects remain unclear, although undoubtedly they will be multiple and complex (Harris-Warrick, 2011). It is likely that each parameter set by an amine – motor burst period, amplitude and stability – involves various cellular targets. For example, all three amines strengthen burst activity, i.e. they increase the amplitude and regularity of bursting by increasing neuronal excitability at the motoneuronal and/or premotoneuronal levels (Berger and Takahashi, 1990; Sqalli-Houssaini and Cazalets, 2000; Kjaerulff and Kiehn, 2001; Gabbay et al., 2002; Zhong et al., 2006b; Han et al., 2007).

Their dose-dependent action on rhythm cycle period (Cazalets et al., 1992; Sqalli-Houssaini and Cazalets, 2000) suggests that the amines directly access components of the spinal CPG itself. Moreover, since EAAs and amines individually increase neuronal excitability, it could be expected that the period of the motor rhythm induced by their combined presence would be even shorter than when each compound is acting separately. This was not the case, and indeed the cycle period was found to be set at an intermediate value between that evoked by EAA or the amine alone (**Figure 1**; **Table 1**). Pharmacological data provide a partial explanation for the aminergic effect on cycle period, since as mentioned previously, both NA and 5-HT slow down the locomotor rhythm through an inhibitory action via α2 receptors (Sqalli-Houssaini and Cazalets, 2000; Gabbay and Lev-Tov, 2004), and 5-HT1 (Beato and Nistri, 1998), while α1, 5-HT7, and 5-HT2 receptor agonists have an activatory action on pattern generation (Cazalets et al., 1992; Sqalli-Houssaini and Cazalets, 2000; Madriaga et al., 2004; Landry et al., 2006; Liu et al., 2009). Surprisingly, however, DA which has been reported solely to have activatory effects also slows down the EAA-induced spinal motor rhythm (**Figure 1**; Barriere et al., 2004). Therefore, it is likely that several underlying processes are operating simultaneously, as reported in other preparations (Harris-Warrick, 2011; Miles and Sillar, 2011; Clemens et al., 2012). For example, in the lamprey the serotonin-induced prolongation of motor burst duration and cycle period involves at least two mechanisms, presynaptic inhibition of glutamate release on CPG neurons (Schwartz et al., 2005) and post-synaptic blockade of sAHP (Wallen et al., 1989; El Manira et al., 1994).

### AMINERGIC SHAPING OF THE SPINAL MOTOR PATTERN

The physiology of locomotion in quadrupeds relies mostly on a simplified dichotomy between dynamic locomotor activity in which only hindlimb movements are considered and postural activity during static tasks. The main characteristics of locomotor-like rhythmic activity recorded in the *in vitro* spinal cord preparation essentially consist of burst cycle period, alternating left/right and flexor/extensor phase relationships, and burst duration. We found here that these parameters were amazingly constant whatever the neuromodulatory condition (**Figures 1** and **4**; **Table 2**). This lack of flexibility may reflect a real functional situation since during ongoing locomotion there is an absolute requirement for fundamental parameters such as right/left movement alternation to be strictly maintained on order preserve balance and speed. Alternatively, the apparent lack of modulator-induced flexibility may be attributable to an *in vitro* bias. In the isolated spinal cord preparation, simultaneous ventral root recordings provide only a global view of various temporally overlapping motor activities and, for example, the slightly differing temporal relationships that exist between various muscle groups, as has been observed with electromyographic recordings in the intact animal (Leblond et al., 2003), may not be evident.

In several systems, interactions between distinct rhythmically active networks have been also described, as for example between walking and swimmeret movements in crustaceans (Cattaert and Clarac, 1983; Chrachri and Neil, 1993), gastric and pyloric motor rhythms in the crustacean stomatogastric system (Bartos et al., 1999; Faumont et al., 2005), trunk and hindlimb activities in Xenopus (Beyeler et al., 2008; Rauscent et al., 2009), locomotion and respiration (Kawahara et al., 1989; Morin and Viala, 2002), scratching and locomotion (Hao et al., 2011), respiration and swallowing (Gestreau et al., 2000). In the newt, axial neuronal networks switch from sequential motor burst propagation to stationary phase-locked activation of the same axial muscles when the animal switches from swimming to quadrupedal locomotion (Delvolve et al., 1997). To date, however, the link between the systems level and the underlying cellular mechanisms has only been established in simpler invertebrate systems in which identified neurons can be individually activated and recorded (Cang and Friesen, 2002; Smarandache et al., 2009). Indeed the pharmacological approach that we have used in the present study also has some limitations, especially when considering the temporal aspects of neuromodulation. During functioning in the intact animal it is likely that descending aminergic pathways are momentarily active whereas the pharmacological protocol used here necessarily consists of tonic, continuous bath-application that in turn leads to the disappearance of the temporal dimension of neuromodulation. A second limitation is the state dependence of the system when several bath-applications are performed on the same spinal cord. Indeed, for the NMA/5-HT combination, we observed in a previous study that successive applications elicited sequences of locomotor-like activity with the same characteristics (Cazalets et al., 1999). Exogenous DA has been shown in other systems to lead to persistent changes in ionic conductances (Rodgers et al., 2011; Krenz et al., 2014), and we also reported in the isolated spinal cord preparation (see Materials and Methods, Barriere et al., 2004) that it may exert long-lasting effects. It is for this reason that DA was always the last drug tested in our experiments and at a much lower concentration than that at which these long-lasting effects were previously observed. There is no evidence that NA exerts such persistent actions.

When considering the integrated functioning of hindlimb and axial networks involved in dynamic posture, a different perspective is required since as we demonstrate, this <<metanetwork>> can be configured into various functional states according to the neuromodulatory environment. As shown in **Figures 1** and **2**, although the amines and EAAs have a qualitatively cumulative effect on rhythm stability and burst amplitude, each amine has its own characteristic imprint. DA and NA specifically enhance sacral motor bursts (**Figure 2**) while 5-HT and DA better stabilize the temporal parameters of the motor pattern (**Figure 1**). Each amine can also specify distinct phase relationships between the various spinal segments (**Figure 3**). In a previous study, in which only the action of a mixture of NMA/5-HT on the interconnected spinal networks was investigated, we found a constant lag between lumbar and thoracic segments, with a significant tendency for caudo-rostral propagation (Falgairolle and Cazalets, 2007). Here, we find that DA and NA, in contrast to 5-HT’s action, substantially decreases the phase lags along the cord so that almost synchronous bursting occurs throughout the spinal cord. Changes from rostro-caudal to caudo-rostral propagation are also observed in the lamprey, when switching from forward to backward swimming occurs (Islam et al., 2006). Although, the cellular targets have not been identified, spinal commissural interneurons could be one possible target as they are known to be modulated by 5-HT (Zhong et al., 2006a), and since we previously established in the neonatal rat that a partial or complete hemicord section suppressed the phase delays between sacral segments (Falgairolle and Cazalets, 2007).

In conclusion, the present paper provides new data on the coordinating processes between spinal cord networks. We demonstrate that each of three amines 5-HT, DA, and NA, when associated with EAA receptor agonists elicits a specific locomotor pattern whose temporal characteristics, motor burst amplitude and intersegmental phase relationships have a distinct signature. These differences are likely to be related to changing behavioral requirements. Furthermore, we find here that the various amines acting in combination do not elicit more robust locomotor-like activity than when bath-applied alone (i.e., more is not better!). The aminergic neuromodulators thus represent an important potential source of adaptive flexibility at the level of the underlying central neuronal networks and the motor output patterns they produce.

## Conflict of Interest Statement

The authors declare that the research was conducted in the absence of any commercial or financial relationships that could be construed as a potential conflict of interest.

## References

[B1] Akazawa K. Aldridge J. W. Steeves J. D. Stein R. B. (1982). Modulation of stretch reflexes during locomotion in the mesencephalic cat. *J. Physiol.* 329 553–567 7143259 10.1113/jphysiol.1982.sp014319 PMC1224796

[B2] Ballion B. Morin D. Viala D. (2001). Forelimb locomotor generators and quadrupedal locomotion in the neonatal rat. *Eur. J. Neurosci.* 14 1727–1738 10.1046/j.0953-816x.2001.01794.x 11860467

[B3] Barriere G. Mellen N. Cazalets J.-R. (2004). Neuromodulation of the locomotor network by dopamine in the isolated spinal cord of newborn rat. *Eur. J. Neurosci.* 19 1325–1335 10.1111/j.1460-9568.2004.03210.x 15016090

[B4] Bartos M. Manor Y. Nadim F. Marder E. Nusbaum M. P. (1999). Coordination of fast and slow rhythmic neuronal circuits. *J. Neurosci.* 19 6650–6660 10414994 10.1523/JNEUROSCI.19-15-06650.1999 PMC6782802

[B5] Beato M. Bracci E. Nistri A. (1997). Contribution of NMDA and non-NMDA glutamate receptors to locomotor pattern generation in the neonatal rat spinal cord. *Proc. Biol. Sci.* 264 877–884 10.1098/rspb.1997.0122 9225479 PMC1688428

[B6] Beato M. Nistri A. (1998). Serotonin-induced inhibition of locomotor rhythm of the rat isolated spinal cord is mediated by the 5-HT1 receptor class. *Proc. Biol. Sci.* 265 2073–2080 10.1098/rspb.1998.0542 9842733 PMC1689497

[B7] Berens P. (2009). CircStat: a MATLAB toolbox for circular statistics. *J. Stat. Softw.* 31 1–21

[B8] Berger A. J. Takahashi T. (1990). Serotonin enhances a low-voltage-activated calcium current in rat spinal motoneurons. *J. Neurosci.* 10 1922–1928 2355258 10.1523/JNEUROSCI.10-06-01922.1990 PMC6570293

[B9] Beyeler A. Metais C. Combes D. Simmers J. Le Ray D. (2008). Metamorphosis-induced changes in the coupling of spinal thoraco-lumbar motor outputs during swimming in *Xenopus laevis*. *J. Neurophysiol.* 100 1372–1383 10.1152/jn.00023.2008 18596184

[B10] Cang J. Friesen W. O. (2002). Model for intersegmental coordination of leech swimming: central and sensory mechanisms. *J. Neurophysiol.* 87 2760–2769 12037178 10.1152/jn.2002.87.6.2760

[B11] Cattaert D. Clarac F. (1983). Influence of walking on swimmeret beating in the lobster *Homarus gammarus*. *J. Neurobiol.* 14 421–439 10.1002/neu.480140603 6644286

[B12] Cazalets J. R. (2000). “Organization of the spinal locomotor network in neonatal rat,” in *Neurobiology of Spinal Cord Injury* eds Kalb R. Stritmatter S. M. (Totowa, NJ: Humana Press Inc.) 89–111 10.1007/978-1-59259-200-5_4

[B13] Cazalets J. R. Grillner P. Menard I. Cremieux J. Clarac F. (1990). Two types of motor rhythm induced by NMDA and amines in an in vitro spinal cord preparation of neonatal rat. *Neurosci. Lett.* 111 116–121 10.1016/0304-3940(90)90354-C 2186309

[B14] Cazalets J. R. Sqalli-Houssaini Y. Clarac F. (1992). Activation of the central pattern generators for locomotion by serotonin and excitatory amino acids in neonatal rat. *J. Physiol.* 455 187–204 1362441 10.1113/jphysiol.1992.sp019296 PMC1175639

[B15] Cazalets J. R. Sqalli-Houssaini Y. Magoul R. (1999). Differential effects of potassium channel blockers on the activity of the locomotor network in neonatal rat. *Brain Res.* 827 185–197 10.1016/S0006-8993(99)01342-6 10320708

[B16] Chrachri A. Neil D. M. (1993). Interaction and synchronization between two abdominal motor systems in crayfish. *J. Neurophysiol.* 69 1373–1383 8389820 10.1152/jn.1993.69.5.1373

[B17] Clemens S. Belin-Rauscent A. Simmers J. Combes D. (2012). Opposing modulatory effects of D1- and D2-like receptor activation on a spinal central pattern generator. *J. Neurophysiol.* 107 2250–2259 10.1152/jn.00366.2011 22262823

[B18] Combes D. Sillar K. T. Simmers J. (2012). A switch in aminergic modulation of locomotor CPG output during amphibian metamorphosis. *Front. Biosci. (Schol. Ed.)* 4:1364–1374 22652878 10.2741/s338

[B19] Delvolve I. Bem T. Cabelguen J. M. (1997). Epaxial and limb muscle activity during swimming and terrestrial stepping in the adult newt, *Pleurodeles waltl*. *J. Neurophysiol.* 78 638–650 9307101 10.1152/jn.1997.78.2.638

[B20] De Sèze M. Falgairolle M. Viel S. Assaiante C. Cazalets J.-R. (2008). Sequential activation of axial muscles during different forms of rhythmic behavior in man. *Exp. Brain Res.* 185 237–247 10.1007/s00221-007-1146-2 17940760

[B21] Dietz V. (2002). Do human bipeds use quadrupedal coordination? *Trends* *Neurosci.* 25 462–467 10.1016/S0166-2236(02)02229-4 12183207

[B22] Duenas S. H. Loeb G. E. Marks W. B. (1990). Monosynaptic and dorsal root reflexes during locomotion in normal and thalamic cats. *J. Neurophysiol.* 63 1467–1476 2358886 10.1152/jn.1990.63.6.1467

[B23] Duysens J. Van de Crommert H. W. (1998). Neural control of locomotion: the central pattern generator from cats to humans. *Gait Posture* 7 131–141 10.1016/S0966-6362(97)00042-8 10200383

[B24] El Manira A. Tegner J. Grillner S. (1994). Calcium-dependent potassium channels play a critical role for burst termination in the locomotor network in lamprey. *J. Neurophysiol.* 72 1852–1861 7823105 10.1152/jn.1994.72.4.1852

[B25] Falgairolle M. Cazalets J. R. (2007). Metachronal coupling between spinal neuronal networks during locomotor activity in newborn rat. *J. Physiol.* 580 87–102 10.1113/jphysiol.2006.115709 17185345 PMC2075426

[B26] Falgairolle M. Ceccato J.-C. Seze M. D. Herbin M. Cazalets J.-R. (2013). Metachronal propagation of motor activity. *Front. Biosci. (Landmark Ed.)* 18:820–837 23747850 10.2741/4146

[B27] Falgairolle M. De Seze M. Juvin L. Morin D. Cazalets J. R. (2006). Coordinated network functioning in the spinal cord: an evolutionary perspective. *J. Physiol. Paris* 100 304–316 10.1016/j.jphysparis.2007.05.003 17658245

[B28] Faumont S. Combes D. Meyrand P. Simmers J. (2005). Reconfiguration of multiple motor networks by short- and long-term actions of an identified modulatory neuron. *Eur. J. Neurosci.* 22 2489–2502 10.1111/j.1460-9568.2005.04442.x 16307592

[B29] Gabbay H. Delvolvé I. Lev-Tov A. (2002). Pattern generation in caudal-lumbar and sacrococcygeal segments of the neonatal rat spinal cord. *J. Neurophysiol.* 88 732–739 12163525 10.1152/jn.2002.88.2.732

[B30] Gabbay H. Lev-Tov A. (2004). Alpha-1 adrenoceptor agonists generate a “fast” NMDA receptor-independent motor rhythm in the neonatal rat spinal cord. *J. Neurophysiol.* 92 997–1010 10.1152/jn.00205.2004 15084642

[B31] Gestreau C. Grelot L. Bianchi A. L. (2000). Activity of respiratory laryngeal motoneurons during fictive coughing and swallowing. *Exp. Brain Res.* 130 27–34 10.1007/s002210050003 10638438

[B32] Gordon I. T. Whelan P. J. (2006). Monoaminergic control of cauda-equina-evoked locomotion in the neonatal mouse spinal cord. *J. Neurophysiol.* 96 3122–3129 10.1152/jn.00606.2006 16956991

[B33] Gramsbergen A. Geisler H. C. Taekema H. Van Eykern L. A. (1999). The activation of back muscles during locomotion in the developing rat. *Brain Res. Dev. Brain Res.* 112 217–228 10.1016/S0165-3806(98)00184-9 9878745

[B34] Grinsted A. Moore J. C. Jevrejeva S. (2004). NPG – Abstract – application of the cross wavelet transform and wavelet coherence to geophysical time series. *Nonlinear Process. Geophys*. 11 561–566 10.5194/npg-11-561-2004

[B35] Guertin P. A. (2009). Recovery of locomotor function with combinatory drug treatments designed to synergistically activate specific neuronal networks. *Curr. Med. Chem.* 16 1366–1371 10.2174/092986709787846541 19355892

[B36] Han P. Nakanishi S. T. Tran M. A. Whelan P. J. (2007). Dopaminergic modulation of spinal neuronal excitability. *J. Neurosci.* 27 13192–13204 10.1523/JNEUROSCI.1279-07.2007 18045913 PMC6673410

[B37] Hao Z. Z. Spardy L. E. Nguyen E. B. Rubin J. E. Berkowitz A. (2011). Strong interactions between spinal cord networks for locomotion and scratching. *J. Neurophysiol.* 106 1766–1781 10.1152/jn.00460.2011 21734103

[B38] Harris-Warrick R. M. (2011). Neuromodulation and flexibility in central pattern generator networks. *Curr. Opin. Neurobiol.* 21 685–692 10.1016/j.conb.2011.05.011 21646013 PMC3171584

[B39] Islam S. S. Zelenin P. V. Orlovsky G. N. Grillner S. Deliagina T. G. (2006). Pattern of motor coordination underlying backward swimming in the lamprey. *J. Neurophysiol.* 96 451–460 10.1152/jn.01277.2005 16772518

[B40] Jamon M. Clarac F. (1998). Early walking in the neonatal rat: a kinematic study. *Behav. Neurosci.* 112 1218–1228 10.1037/0735-7044.112.5.1218 9829799

[B41] Jiang Z. Carlin K. P. Brownstone R. M. (1999). An in vitro functionally mature mouse spinal cord preparation for the study of spinal motor networks. *Brain Res.* 816 493–499 10.1016/S0006-8993(98)01199-8 9878874

[B42] Juvin L. Simmers J. Morin D. (2005). Propriospinal circuitry underlying interlimb coordination in mammalian quadrupedal locomotion. *J. Neurosci.* 25 6025–6035 10.1523/JNEUROSCI.0696-05.2005 15976092 PMC6724791

[B43] Kawahara K. Nakazono Y. Yamauchi Y. Miyamoto Y. (1989). Coupling between respiratory and locomotor rhythms during fictive locomotion in decerebrate cats. *Neurosci. Lett.* 103 326–330 10.1016/0304-3940(89)90121-3 2510092

[B44] Kiehn O. Kjaerulff O. (1996). Spatiotemporal characteristics of 5-HT and dopamine-induced rhythmic hindlimb activity in the in vitro neonatal rat. *J. Neurophysiol.* 75 1472–1482 8727391 10.1152/jn.1996.75.4.1472

[B45] Kiehn O. Sillar K. T. Kjaerulff O. Mcdearmid J. R. (1999). Effects of noradrenaline on locomotor rhythm-generating networks in the isolated neonatal rat spinal cord. *J. Neurophysiol.* 82 741–746 10444672 10.1152/jn.1999.82.2.741

[B46] Kjaerulff O. Kiehn O. (2001). 5-HT modulation of multiple inward rectifiers in motoneurons in intact preparations of the neonatal rat spinal cord. *J. Neurophysiol.* 85 580–593 11160495 10.1152/jn.2001.85.2.580

[B47] Koehler W. J. Schomburg E. D. Steffens H. (1984). Phasic modulation of trunk muscle efferents during fictive spinal locomotion in cats. *J. Physiol.* 353 187–197 6237190 10.1113/jphysiol.1984.sp015331 PMC1193302

[B48] Krenz W. D. Parker A. R. Rodgers E. W. Baro D. J. (2014). Dopaminergic tone persistently regulates voltage-gated ion current densities through the D1R-PKA axis, RNA polymerase II transcription, RNAi, mTORC 1 and translation. *Front. Cell. Neurosci.* 8:39 10.3389/fncel.2014.00039 PMC3925969 24596543

[B49] Kudo N. Yamada T. (1987). N-methyl-D,L-aspartate-induced locomotor activity in a spinal cord-hindlimb muscles preparation of the newborn rat studied in vitro. *Neurosci. Lett.* 75 43–48 10.1016/0304-3940(87)90072-3 3554010

[B50] Landry E. S. Lapointe N. P. Rouillard C. Levesque D. Hedlund P. B. Guertin P. A. (2006). Contribution of spinal 5-HT1A and 5-HT7 receptors to locomotor-like movement induced by 8-OH-DPAT in spinal cord-transected mice. *Eur. J. Neurosci.* 24 535–546 10.1111/j.1460-9568.2006.04917.x 16836640

[B51] Lapointe N. P. Guertin P. A. (2008). Synergistic effects of D1/5 and 5-HT1A/7 receptor agonists on locomotor movement induction in complete spinal cord-transected mice. *J. Neurophysiol.* 100 160–168 10.1152/jn.90339.2008 18480366

[B52] Lapointe N. P. Rouleau P. Ung R. V. Guertin P. A. (2009). Specific role of dopamine D1 receptors in spinal network activation and rhythmic movement induction in vertebrates. *J. Physiol.* 587 1499–1511 10.1113/jphysiol.2008.166314 19204052 PMC2678221

[B53] Lapointe N. P. Ung R. V. Rouleau P. Guertin P. A. (2008). Effects of spinal alpha(2)-adrenoceptor and I(1)-imidazoline receptor activation on hindlimb movement induction in spinal cord-injured mice. *J. Pharmacol. Exp. Ther.* 325 994–1006 10.1124/jpet.107.134874 18364473

[B54] Leblond H. L’esperance M. Orsal D. Rossignol S. (2003). Treadmill locomotion in the intact and spinal mouse. *J. Neurosci.* 23 11411–11419 14673005 10.1523/JNEUROSCI.23-36-11411.2003 PMC6740531

[B55] Liu J. Akay T. Hedlund P. B. Pearson K. G. Jordan L. M. (2009). Spinal 5-HT7 receptors are critical for alternating activity during locomotion: in vitro neonatal and in vivo adult studies using 5-HT7 receptor knockout mice. *J. Neurophysiol.* 102 337–348 10.1152/jn.91239.2008 19458153

[B56] Maclellan M. J. Ivanenko Y. P. Cappellini G. Sylos Labini F. Lacquaniti F. (2011). Features of hand-foot crawling behavior in human adults. *J. Neurophysiol.* 107 114–125 10.1152/jn.00693.2011 21975454

[B57] Madriaga M. A. Mcphee L. C. Chersa T. Christie K. J. Whelan P. J. (2004). Modulation of locomotor activity by multiple 5-HT and dopaminergic receptor subtypes in the neonatal mouse spinal cord. *J. Neurophysiol.* 92 1566–1576 10.1152/jn.01181.2003 15163678

[B58] Miles G. B. Sillar K. T. (2011). Neuromodulation of vertebrate locomotor control networks. *Physiology (Bethesda)* 26 393–411 10.1152/physiol.00013.2011 22170958

[B59] Mor Y. Lev-Tov A. (2007). Analysis of rhythmic patterns produced by spinal neural networks. *J. Neurophysiol.* 98 2807–2817 10.1152/jn.00740.2007 17715187

[B60] Morin D. Viala D. (2002). Coordinations of locomotor and respiratory rhythms in vitro are critically dependent on hindlimb sensory inputs. *J. Neurosci.* 22 4756–4765 12040083 10.1523/JNEUROSCI.22-11-04756.2002 PMC6758812

[B61] Nakajima T. Mezzarane R. A. Klarner T. Barss T. S. Hundza S. R. Komiyama T. (2013). Neural mechanisms influencing interlimb coordination during locomotion in humans: presynaptic modulation of forearm H-reflexes during leg cycling. *PLoS ONE* 8:e76313 10.1371/journal.pone.0076313 PMC3799938 24204611

[B62] Pearlstein E. Ben Mabrouk F. Pflieger J. F. Vinay L. (2005). Serotonin refines the locomotor-related alternations in the in vitro neonatal rat spinal cord. *Eur. J. Neurosci.* 21 1338–1346 10.1111/j.1460-9568.2005.03971.x 15813943

[B63] Rauscent A. Einum J. Le Ray D. Simmers J. Combes D. (2009). Opposing aminergic modulation of distinct spinal locomotor circuits and their functional coupling during amphibian metamorphosis. *J. Neurosci.* 29 1163–1174 10.1523/JNEUROSCI.5255-08.2009 19176825 PMC6665137

[B64] Rodgers E. W. Krenz W. D. Baro D. J. (2011). Tonic dopamine induces persistent changes in the transient potassium current through translational regulation. *J. Neurosci.* 31 13046–13056 10.1523/JNEUROSCI.2194-11.2011 21917788 PMC3544522

[B65] Rossignol S. (1996). “Neural control of stereotypic limb movements,” in *Handbook of Physiology, Section 12. Exercise: Regulation and Integration of Multiple Systems* eds Rowell B. Sheperd J. T. (Bethesda, MD: American Physiological Society) 173–216

[B66] Schwartz E. J. Gerachshenko T. Alford S. (2005). 5-HT prolongs ventral root bursting via presynaptic inhibition of synaptic activity during fictive locomotion in lamprey. *J. Neurophysiol.* 93 980–988 10.1152/jn.00669.2004 15456802

[B67] Smarandache C. Hall W. M. Mulloney B. (2009). Coordination of rhythmic motor activity by gradients of synaptic strength in a neural circuit that couples modular neural oscillators. *J. Neurosci.* 29 9351–9360 10.1523/JNEUROSCI.1744-09.2009 19625525 PMC2732425

[B68] Smith J. C. Feldman J. L. (1987). In vitro brainstem-spinal cord preparations for study of motor systems for mammalian respiration and locomotion. *J. Neurosci. Methods* 21 321–333 10.1016/0165-0270(87)90126-9 2890797

[B69] Somjen G. G. (1979). Extracellular potassium in the mammalian central nervous system. *Annu. Rev. Physiol.* 41 159–177 10.1146/annurev.ph.41.030179.001111 373587

[B70] Sqalli-Houssaini Y. Cazalets J. R. (2000). Noradrenergic control of locomotor networks in the in vitro spinal cord of the neonatal rat. *Brain Res.* 852 100–109 10.1016/S0006-8993(99)02219-2 10661501

[B71] Sqalli-Houssaini Y. Cazalets J. R. Clarac F. (1993). Oscillatory properties of the central pattern generator for locomotion in neonatal rats. *J. Neurophysiol.* 70 803–813 8410173 10.1152/jn.1993.70.2.803

[B72] Sqalli-Houssaini Y. Cazalets J. R. Fabre J. C. Clarac F. (1991). A cooling/heating system for use with in vitro preparations: study of temperature effects on newborn rat rhythmic activities. *J. Neurosci. Methods* 39 131–139 10.1016/0165-0270(91)90079-F 1798343

[B73] Talpalar A. E. Kiehn O. (2010). Glutamatergic mechanisms for speed control and network operation in the rodent locomotor CPG. *Front. Neural Circuits* 4:19 10.3389/fncir.2010.00019 PMC2938926 20844601

[B74] Thorstensson A. Carlson H. Zomlefer M. R. Nilsson J. (1982). Lumbar back muscle activity in relation to trunk movements during locomotion in man. *Acta Physiol. Scand.* 116 13–20 10.1111/j.1748-1716.1982.tb10593.x 7158389

[B75] Torrence C. Compo G. P. (2010). A practical guide to wavelet analysis. *Bull. Am. Meteorol. Soc.* 79 61–78 10.1175/1520-0477(1998)079&lt;0061:APGTWA&gt;2.0.CO;2

[B76] Wallen P. Christenson J. Brodin L. Hill R. Lansner A. Grillner S. (1989). Mechanisms underlying the serotonergic modulation of the spinal circuitry for locomotion in lamprey. *Prog. Brain Res.* 80 321–327; discussion 315–329 10.1016/S0079-6123(08)62227-X 2699371

[B77] Wannier T. Bastiaanse C. Colombo G. Dietz V. (2001). Arm to leg coordination in humans during walking, creeping and swimming activities. *Exp. Brain Res.* 141 375–379 10.1007/s002210100875 11715082

[B78] Whelan P. Bonnot A. O’donovan M. J. (2000). Properties of rhythmic activity generated by the isolated spinal cord of the neonatal mouse. *J. Neurophysiol.* 84 2821–2833 11110812 10.1152/jn.2000.84.6.2821

[B79] Zehr E. P. Duysens J. (2004). Regulation of arm and leg movement during human locomotion. *Neuroscientist* 10 347–361 10.1177/1073858404264680 15271262

[B80] Zhong G. Diaz-Rios M. Harris-Warrick R. M. (2006a). Intrinsic and functional differences among commissural interneurons during fictive locomotion and serotonergic modulation in the neonatal mouse. *J. Neurosci.* 26 6509–6517 10.1523/JNEUROSCI.1410-06.2006 16775138 PMC6674024

[B81] Zhong G. Diaz-Rios M. Harris-Warrick R. M. (2006b). Serotonin modulates the properties of ascending commissural interneurons in the neonatal mouse spinal cord. *J. Neurophysiol.* 95 1545–1555 10.1152/jn.01103.2005 16338993

[B82] Zhong G. Masino M. A. Harris-Warrick R. M. (2007). Persistent sodium currents participate in fictive locomotion generation in neonatal mouse spinal cord. *J. Neurosci.* 27 4507–4518 10.1523/JNEUROSCI.0124-07.2007 17460064 PMC6673000

[B83] Zomlefer M. R. Provencher J. Blanchette G. Rossignol S. (1984). Electromyographic study of lumbar back muscles during locomotion in acute high decerebrate and in low spinal cats. *Brain Res.* 290 249–260 10.1016/0006-8993(84)90942-9 6692142

